# Regional Disparities in the Use and Demand for Digital Health Services for Autism Spectrum Disorder in China: Cross-Sectional Survey of Stakeholder Perspectives

**DOI:** 10.2196/77157

**Published:** 2025-10-03

**Authors:** Mingyang Zou, Xiaomei Gong, Liwen Feng, Shengqi Li, Chenyang Lu, Zhuoqiong Liu, Caihong Sun, Lijie Wu

**Affiliations:** 1 Department of Children’s and Adolescent Health Public Health College Harbin Medical University Harbin China; 2 Heilongjiang Province Key Laboratory of Child Development and Genetic Research Harbin Medical University Harbin China; 3 Beijing Normal—Hong Kong Baptist University Zhuhai China; 4 Department of Developmental Behavioral Pediatrics The Sixth Affiliated Hospital Harbin Medical University Harbin China

**Keywords:** autism spectrum disorder, digital health, caregivers, rehabilitation therapists, China

## Abstract

**Background:**

Autism spectrum disorder (ASD) is a lifelong neurodevelopmental condition, the prevalence of which is increasing in China and worldwide. Digital health technologies offer promising solutions for improving screening, diagnosis, and rehabilitation of children with ASD, particularly in resource-limited settings. However, digital health technologies for ASD have not been adopted in China. Understanding utilization patterns, influencing factors, and user needs is essential to inform equitable, effective digital health strategies.

**Objective:**

This study aimed to assess the current use, influencing factors, and perceived needs of digital health services among parents of children with ASD and rehabilitation therapists in 2 distinct provinces in China.

**Methods:**

A cross-sectional survey was carried out between November 2023 and February 2024 in Heilongjiang and Fujian provinces. Purposive sampling recruited a total of 780 parents and 745 rehabilitation therapists to complete a structured questionnaire. Data were analyzed using descriptive statistics, stepwise multivariable logistic regression, and multiple response analysis.

**Results:**

The use of digital health services was low among parents (46/780, 5.9%) and rehabilitation therapists (161/745, 21.6%), although the demand was substantially higher (621/780, 79.6% and 671/745, 90.0%, respectively). Among parents, higher use was positively associated with younger age (20-29 years), employment, lower income (≤CNY 3000/month [US $421.247/month]), and delayed ASD diagnosis (7-12 years old). Among rehabilitation therapists, male, special education background, and autism-specific training experience predicted higher use (all *P*<.05). Demand was significantly greater in Fujian than in Heilongjiang and was positively associated with higher education levels in parents and rehabilitation therapists (*P*<.05). Parents and rehabilitation therapists valued a greater understanding of ASD behaviors and development and easier access to resources as primary benefits. Common barriers to the use of digital health services included high cost, need for additional equipment, and usability challenges. The smartphone-WeChat mini-program was the preferred device and platform, respectively. Parents prioritized access to rehabilitation courses, remote guidance, and policy information, while rehabilitation therapists favored personalized plans and professional skills training.

**Conclusions:**

Despite a strong demand, digital health services are underused in ASD care across China, with adoption of digital health services influenced by regional disparities and sociodemographic factors. This study, as the first comparative analysis of parents and rehabilitation therapists in 2 Chinese provinces, provides stakeholder-specific insights to guide targeted, locally relevant interventions. Bridging the digital divide through inclusive policies, training, and cross-sector collaboration will be essential for equitable integration into ASD care pathways.

## Introduction

Autism spectrum disorder (ASD) is a lifelong neurodevelopmental condition that significantly impacts an individual’s quality of life. The global prevalence of ASD has risen markedly in recent decades, reaching approximately 1.0% worldwide and 0.7% among Chinese children, according to recent epidemiologic studies [1,2]. Currently, there are no pharmacologic treatments specifically approved for ASD, making early detection and timely, evidence-based intervention critical for improving long-term outcomes and mitigating symptom progression [3]. However, early screening and diagnosis are challenging. Existing diagnostic procedures often require parents to complete extensive questionnaires, followed by in-person clinical evaluations, a process that is time-consuming and resource-intensive. These challenges are further exacerbated in rural or socioeconomically disadvantaged areas with limited access to specialized health care resources [4]. As a result, significant diagnostic delays persist. Studies have shown that up to 25% of children with ASD are not identified until they begin formal schooling, with the average age of diagnosis being approximately 6 years [5,6]. This finding underscores the urgent need for scalable, efficient, and objective screening solutions. Technological innovation, particularly digital health, offers a promising pathway to streamline ASD diagnosis, reduce the clinical burden, and improve early detection. Moreover, the growing ASD population highlights the need for expanded access to cost-effective, personalized therapeutic interventions. Traditional behavioral therapies, which rely heavily on trained specialists in clinical settings, are often costly and inaccessible, especially given ongoing shortages of qualified therapists [7]. The COVID-19 pandemic further disrupted face-to-face services while simultaneously accelerating the global adoption of digital tools in health care, education, and research [8].

Digital health technologies, as defined by the World Health Organization, involve the use of digital and smart technologies, including artificial intelligence (AI), the internet of things, big data, robotics, and connected devices, to improve health outcomes [9]. These technologies offer significant advantages with respect to efficiency, scalability, and user accessibility. For example, mobile machine-learning apps, like Cognoa, allow parents to effectively screen children between 18 and 72 months of age for ASD risk within minutes at home, thereby enabling rapid triage and early intervention [10]. Similarly, a mobile app (Autism AI), has simplified the ASD screening process by enabling caregivers and family members to easily access assessment tools that deliver immediate results with high sensitivity and specificity. Autism AI uses machine learning algorithms that continuously update the models based on new population data, which improves adaptability across different developmental stages [11]. Digital health also shows promise in ASD rehabilitation. By leveraging virtual reality (VR) and augmented reality (AR) technologies, digital health provides a more immersive and interactive rehabilitation experience. These technologies enable the delivery of personalized interventions at home and in clinical settings, while enhancing patient engagement and improving therapeutic outcomes through dynamic and engaging treatment environments. Compared with traditional therapies, these tools better simulate real-world social scenarios, enhancing social cognition and functional skills. For example, gamified learning can teach children with ASD how to take the bus, cross the street, and shop independently [12,13]. Remote interventions via earning web-based platforms further extend the reach of these interventions to underserved areas [14]. Robotic systems are another emerging modality in ASD care. Humanoid robots such as NAO have been shown to improve attention, motor skills, and social engagement in children with ASD, especially those who struggle with direct human interaction [15]. These tools facilitate group-based interventions and remote therapy, easing the logistical burden on families and clinicians.

Despite the promise of digital health services, the development and application of digital health services for ASD are limited in China. Interdisciplinary collaboration among policy makers, health care practitioners, technology developers, and end users is crucial to ensure that these technological solutions align with the needs of individuals with ASD and their caregivers. Therefore, this study aims to examine the current use and perceived needs of digital health services among parents and rehabilitation professionals caring for individuals with ASD in China, and to assess perceived benefits, barriers, and preferences related to digital health services. The findings will help guide future research in the design of digital health technologies as well as policy makers.

## Methods

### Study Design and Participants

This cross-sectional study was conducted between November 2023 and February 2024 in 2 geographically distinct provinces in China (Heilongjiang [northern region] and Fujian [southern region]). Purposive sampling was used to enroll participants who satisfied the following criteria: (1) parents/primary caregivers of children 0-18 years of age with a confirmed ASD diagnosis according to DSM-5 criteria who could understand Chinese and complete the questionnaire independently; and (2) rehabilitation therapists, including special education teachers, psychologists, and rehabilitation nurses, currently providing ASD-related intervention services, with at least 6 months of professional experience in the field [16]. Participants were recruited from designated rehabilitation institutions affiliated with the Heilongjiang Disabled Persons’ Federation, the Fujian Disabled Persons’ Federation, the Rehabilitation Department of Fujian Children’s Hospital, and the Developmental Behavioral Pediatrics Department of Xiamen Maternal and Child Health Hospital. Each of these institutions specializes in pediatric developmental disorders and provides rehabilitation services for children with ASD.

### Questionnaire Content

A structured, self-administered questionnaire was designed in collaboration with a multidisciplinary expert panel, including clinical specialists and nursing staff, to ensure relevance and comprehensive coverage of digital health domains. Content validity was verified through a preliminary pilot test. Two tailored versions of the questionnaire were developed: for parents and rehabilitation professionals (Multimedia Appendix 1 contains details for the full instrument). The questionnaire consisted of 2 sections.

Section 1 elicits demographic and background information. Specifically, Section 1 elicited the following information from parents: demographic variables (province, sex, age, and household registration); socioeconomic indicators (education level and employment status); child-specific factors (child’s age, sex, age at diagnosis, and symptom severity); family background (marital status, number of children, and monthly household income); and rehabilitation-related characteristics (monthly rehabilitation expenses, cumulative rehabilitation duration, and years spent caregiving). In addition, Section 1 elicited the following information from therapists: demographic information (province, sex, and age); socioeconomic status (education level and specialty); and work experience (autism-specific skills training and years in practice).

Section 2 determined digital health use and needs assessment. Specifically, section 2 evaluated the following 6 dimensions of digital health services: current usage status, perceived benefits, perceived obstacles, preferred devices, technologies, and content requirements.

### Procedures

Data were collected via a web-based platform (Questionnaire Star; Changsha Ranxing Science and Technology Co, Ltd). Participants accessed the survey through unique QR codes embedded in electronic invitations. The recruitment procedures were as follows. (1) The research team collaborated with each institution to identify potential participants during routine clinical visits or rehabilitation sessions. (2) On-site staff, including pediatricians, rehabilitation therapists, and institutional administrators, verbally informed eligible individuals about the study purpose, requirements, and confidentiality measures. (3) Interested participants received a unique QR code generated using the Questionnaire Star platform via printed flyers or direct digital sharing (eg, through WeChat). (4) For parent participants, distribution occurred primarily in waiting areas of outpatient clinics or rehabilitation centers during their child’s visit. (5) For therapists, QR codes were shared via institutional workgroups or during staff meetings. (6) Participants scanned the QR codes to access the web-based informed consent form, and only those participants who provided consent proceeded to complete the questionnaire. Further details can be found on the Checklist for Reporting Results of Internet E-Surveys (CHERRIES) listed in Multimedia Appendix 2.

To ensure data quality, responses were anonymized, all items were required, and restrictions were placed to prevent duplicate submissions from the same IP address. Invalid questionnaires were excluded based on the following criteria: (1) patterned or identical responses (eg, all “1” or sequential “1, 2, 3, 4”); (2) logical inconsistencies; or (3) completion time in <2 min. Of the 906 parental and 841 therapist responses initially received, 126 and 96 were excluded, respectively, resulting in valid response rates of 86.09% (parents) and 88.58% (therapists). The reliability of the instruments was high, with Cronbach α coefficients of 0.94 for the parent version and 0.96 for the therapist version.

### Statistical Analysis

Statistical analyses were performed using SPSS (version 27; IBM Corp). Descriptive statistics were presented as frequencies and percentages for categorical variables. Group differences in categorical variables are assessed using chi-square tests. The multiple-choice question response analysis was performed using the multiple-response analysis method. To identify factors associated with the use and demand for digital health services, univariant and stepwise multivariable logistic regression analyses were performed. The use and perceived need of digital health services were designated dependent variables. Relevant demographic and background characteristics specific to each participant group were designated independent variables. The odds ratios (ORs) and 95% CIs were calculated. A forest plot was constructed using R software (version 4.4.1; Lucent Technologies). Bar and line charts were plotted using GraphPad (GraphPad Software, LLC) Prism. All statistical tests were 2-sided, at a *P* value of <.05.

### Ethical Consideration

This study was conducted in strict adherence to the ethical standards set forth in the Declaration of Helsinki, and was approved (LC2024-066) by the Institutional Review Board of the Sixth Affiliated Hospital, Harbin Medical University (Harbin, China). All procedures were carried out with an adequate understanding by participants, and each participant provided web-based informed consent prior to commencing the study. Participation was voluntary, and no incentives were provided for survey completion. All collected data were kept strictly confidential and anonymous.

## Results

### Sample Characteristics

A total of 780 parents of children with ASD and 745 rehabilitation therapists participated in the study. The demographic and background information of the parents are presented in Table 1. There were significant differences between participants from Fujian and Heilongjiang provinces with respect to household registration, employment status, monthly household income, child’s age, age at the time of diagnosis, cumulative rehabilitation duration, and years spent caregiving (*P*=.03 *P*<.001, *P*<.001, *P*<.001, *P*=.03, *P*<.001, and *P*<.001, respectively). The demographic characteristics of rehabilitation therapists are shown in Table 2. Statistically significant differences were detected in the education level between provinces (*P*=.03). Notably, the proportion of therapists in Fujian who had received autism-specific skills training was significantly higher than in Heilongjiang (*P*=.009).

**Table 1:** Demographic characteristics of parents of children with autism spectrum disorder (n=780).

Characteristics		Heilongjiang, n (%)	Fujian, n (%)	Chi-square (*df*)		*P* value	
**Sex**					3.358 (1)		.07
	Male	100 (14.9)	24 (21.8)				
	Female	570 (85.1)	86 (78.2)				
**Age (years)**					2.147 (1)		.14
	20-29	50 (7.5)	4 (3.6)				
	≥30	620 (92.5)	106 (96.4)				
**Education level**					4.524 (2)		.10
	University and above	161 (24.0)	34 (30.9)				
	Vocational college	163 (24.3)	31 (28.2)				
	High school and below	346 (51.7)	45 (40.9)				
**Household registration**					4.966 (1)		.03
	Urban	356 (53.1)	71 (64.5)				
	Rural	314 (46.9)	39 (35.5)				
**Employment status**					32.893 (1)		<.001
	Employed	284 (42.2)	79 (71.8)				
	Unemployed	386 (57.6)	31 (28.2)				
**Marital status**					4.604 (3)		.20
	Married	569 (84.9)	100 (90.9)				
	Divorced	50 (7.5)	4 (3.6)				
	Widowed	6 (0.9)	2 (1.8)				
	Separated	45 (6.7)	4 (3.6)				
**Monthly household income (US $1=CNY 7.1217)**					35.687 (1)		<.001
	≤3000	216 (32.2)	5 (4.5)				
	>3000	454 (67.8)	105 (95.5)				
**Monthly rehabilitation costs (US $1=CNY 7.1217)**					1.998 (1)		.16
	≤3000	235 (35.1)	31 (28.2)				
	>3000	435 (64.9)	79 (71.8)				
**Child’s sex**					0.276 (1)		.60
	Male	503(75.1)	80(72.7)				
	Female	167(24.9)	30(27.3)				
**Child’s age (years)**					41.765 (3)		<.001
	≥13	10 (1.5)	13 (11.8)				
	7-12	173 (25.8)	38 (34.5)				
	4-6	378 (56.4)	45 (40.9)				
	0-3	109 (16.3)	14 (12.7)				
**Symptom severity**					2.764 (3)		.43
	Mild	199 (29.7)	37 (33.6)				
	Moderate	262 (39.1)	41 (37.3)				
	Severe	108 (16.1)	21 (19.1)				
	Unclear	101 (15.1)	11 (10.0)				
**Age at diagnosis (years)**					7.001 (2)		.03
	7-12	3 (0.4)	0 (0.0)				
	4-6	179 (26.7)	17 (15.5)				
	0-3	488 (72.8)	93 (84.5)				
**Cumulative rehabilitation duration (years)**					42.299 (3)		<.001
	＜1	140 (20.9)	14 (12.7)				
	1-2	301 (44.9)	37 (33.6)				
	3-4	164 (24.5)	24 (21.8)				
	≥5	65 (9.7)	35 (31.8)				
**Years spent caregiving (years)**					40.496 (3)		<.001
	≥5	116 (17.3)	48 (43.6)				
	3-4	182 (27.2)	17 (15.5)				
	1-2	175 (26.1)	24 (21.8)				
	＜1	197 (29.4)	21 (19.1)				

**Table 2:** Demographic characteristics of rehabilitation therapists with autism spectrum disorder–related experiences (n=745).

Characteristics			Heilongjiang, n (%)		Fujian, n (%)		Chi-square (*df*)			*P* value	
**Sex**								0.185 (1)			.67
	Male	62 (11.0)		22 (12.2)							
	Female	502 (89.0)		159 (87.8)							
**Age (years)**								0.711 (1)			.40
	20-29	168 (29.8)		48 (26.5)							
	≥30	396 (70.2)		133 (73.5)							
**Education level**								7.158 (2)			.03
	University and above	287 (50.9)		74 (40.9)							
	Vocational college	223 (39.5)		80 (44.2)							
	High school and below	54 (9.6)		27 (14.9)							
**Specialty**								6.545 (3)			.09
	Special education	111 (19.7)		28 (15.5)							
	Preschool education	154 (27.3)		59 (32.6)							
	Rehabilitation medicine, psychology, and nursing	190 (33.7)		49 (27.1)							
	Others	109 (19.3)		45 (24.9)							
**Autism-specific skills training**								6.816 (1)			.009
	Yes	498 (88.3)		146 (80.7)							
	No	66 (11.7)		35 (19.3)							
**Years in practice**								7.389 (3)			.06
	＜1 year	107 (19.0)		33 (18.2)							
	1-5 years	248 (44.0)		99 (54.7)							
	6-10 years	122 (21.6)		28 (15.5)							
	≥10 years	87 (15.4)		21 (11.6)							

### Use of Digital Health Services

The overall digital health services utilization rate among parents was 5.9% (46/780) with provincial rates of 6.1% (41/670) in Heilongjiang and 4.5% (5/110) in Fujian (*χ*^2^_1_=0.422, *P*=.52; Table S1 in Multimedia Appendix 3). Among rehabilitation therapists, the overall utilization rate was 21.6% with similar rates in Heilongjiang (121/564, 21.5%) and Fujian (40/181, 22.1%; *χ*^2^_1_=0.034, *P*=.85; Table S2 in Multimedia Appendix 3). A multiple stepwise logistic regression was performed to identify factors associated with the use of digital health services. The dependent variable was defined as use (1) or nonuse (0) of digital health services, with all other factors entered as independent variables.

Logistic regression analyses showed that parental use of digital health services was associated with age, employment status, monthly household income, and the child’s age at the time of diagnosis. Specifically, parents between 20 and 29 years of age were 4.286 times more likely to use digital health services than parents ≥30 years of age (OR 4.286, 95% CI 1.743-10.541). Employment was a positive predictor of digital health services use (OR 3.140, 95% CI 1.605-6.146). Parents with an average monthly household income ≤CNY 3000 (US $1=CNY 7.1217) were more likely to use digital health tools than parents with an average monthly household income >CNY 3000 (US $1=CNY 7.1217; OR 2.927, 95% CI 1.515-5.653). Parents whose children were between 7 and 12 years of age at the time of ASD diagnosis were associated with greater use compared with parents whose children were between 0 and 3 years of age at the time of ASD diagnosis (OR 16.997, 95% CI 1.395-207.171; Table 3).

**Table 3:** Multivariable logistic regression analyses (stepwise) for estimating determinants of digital health service use among parents and rehabilitation therapists caring for individuals with autism spectrum disorder based on a cross-sectional survey conducted in Heilongjiang and Fujian provinces, China.

Characteristics				Use, n (%)		Nonuse, n (%)		OR^a^ (95% CI)		*P* value
**Parents**										
	**Age (years)**									
		≥30	38 (5.2)		688 (94.8)		—^b^		—	
		20-29	8 (14.8)		46 (85.2)		4.286 (1.743-10.541)		.002	
	**Employment status**									
		Unemployed	16 (3.8)		401 (96.2)		—		—	
		Employed	30 (8.3)		333 (91.7)		3.140 (1.605-6.146)		<.001	
	**Monthly household income (US $1=CNY 7.1217)**									
		＞3000	26 (4.7)		533 (95.3)		—		—	
		≤3000	20 (9.0)		201 (91.0)		2.927 (1.515-5.653)		.001	
	**Age at diagnosis (years)**									
		0-3	31 (5.3)		550 (94.7)		—		—	
		4-6	14 (7.1)		182 (92.9)		1.519 (0.767-3.007)		.23	
		7-12	1 (33.3)		2 (66.7)		16.997 (1.395-207.171)		.03	
	**Years spent caregiving (years)**									
		≥5	8 (4.9)		156 (95.1)		—		—	
		3-4	19 (9.5)		180 (90.5)		2.405 (0.995-5.811)		.05	
		1-2	11 (5.5)		188 (94.5)		1.050 (0.396-2.779)		.92	
		＜1	8 (3.7)		210 (96.3)		0.539 (0.187-1.555)		.25	
**Rehabilitation therapists**										
	**Sex**									
		Female	133 (20.1)		528 (79.9)		—		—	
		Male	28 (33.3)		56 (66.7)		2.057 (1.224-3.4580)		.006	
	**Specialty**									
		Others	24 (15.6)		130 (84.4)		—		—	
		Special education	45 (32.4)		94 (67.6)		2.331 (1.310-4.150)		.004	
		Preschool education	43 (20.2)		170 (79.8)		1.308 (0.741-2.307)		.35	
		Rehabilitation medicine, psychology, and nursing	49 (20.5)		190 (79.5)		1.341 (0.774-2.324)		.30	
	**Autism-specific skills training**									
		No	4 (4.0)		97 (96.0)		—		—	
		Yes	157 (24.4)		487 (75.6)		7.124 (2.561-19.815)		<.001	

^a^OR: odds ratio.

^b^Reference values.

Logistic regression analyses indicated that male rehabilitation therapists were 2.057 times more likely to use digital health services than females (OR 2.057, 95% CI 1.224-3.458). The rehabilitation therapists specializing in special education had higher use of digital health services than rehabilitation therapists who did not specialize in special education (OR 2.331, 95% CI 1.310-4.150). Rehabilitation therapists who had received autism-specific skills training were substantially more likely to use digital health services than rehabilitation therapists who had not received such training (OR 7.124, 95% CI 2.561-19.815; Table 3).

### Demand for Digital Health Services

The overall demand for digital health services was 79.6% (621/780) among parents, with the rate in Heilongjiang (521/670, 77.8%) significantly lower than in Fujian (100/110, 90.9%; *χ*^2^_1_=10.064, *P*=.002; Table S3 in Multimedia Appendix 3). Similarly, 90.0% of rehabilitation therapists reported a demand for digital health services with a significantly lower rate in Heilongjiang (498/564, 88.3%) compared with Fujian (170/181, 93.9%; *χ*^2^_1_=4.678, *P*=.03; Table S4 in Multimedia Appendix 3). Stepwise logistic regression was also performed to identify factors associated with the demand for digital health services. The dependent variable was defined as no demand (0) or presence of a demand (1) for digital health services, with all other factors entered as independent variables.

Logistic regression analyses showed that parental use of digital health services was significantly associated with province and education level. Parents residing in Fujian were significantly more likely to express demand for digital health services than parents residing in Heilongjiang (OR 2.750, 95% CI 1.373-5.509). Higher education level was positively associated with demand. Specifically, parents with a university degree or higher were nearly 2.993 times more likely to express a demand for digital health services than parents with a high school education or below (OR 2.993, 95% CI 1.778-5.039; Table 4).

Rehabilitation therapists from Fujian reported a greater demand for digital health services than parents in Heilongjiang (OR 2.362, 95% CI 1.203-4.639). Rehabilitation therapists with vocational or university-level education were significantly more likely to express a demand for digital health services than rehabilitation therapists with a high school diploma or below (OR 2.777, 95% CI 1.465-5.264 and OR 4.415, 95% CI 2.278-8.556, respectively; Table 4).

**Table 4:** Multivariable logistic regression analyses (stepwise) for estimating determinants of demand for digital health services among parents and rehabilitation therapists caring for individuals with autism spectrum disorder based on a cross-sectional survey conducted in Heilongjiang and Fujian provinces, China.

Characteristics			Demand, n (%)	No demand, n (%)	OR^a^ (95% CI)	*P* value
**Parents**						
	**Province**					
		Heilongjiang	521 (77.8)	149 (22.2)	—^b^	—
		Fujian	100 (90.9)	10 (9.1)	2.750 (1.373-5.509)	.004
	**Education level**					
		High school and below	291 (74.4)	100 (25.6)	—	—
		Vocational college	155 (79.9)	39 (20.1)	1.342 (0.878-2.053)	.17
		University and above	175 (89.7)	20 (10.3)	2.993 (1.778-5.039)	<.001
	**Years spent caregiving**					
		≥5 years	132 (80.5)	32 (19.5)	—	—
		3-4 years	164 (82.4)	35 (17.6)	1.386 (0.800-2.402)	.25
		1-2 year	166 (83.4)	33 (16.6)	1.427 (0.820-2.484)	.21
		＜1 year	159 (72.9)	59 (27.1)	0.750 (0.451-1.246)	.27
**Rehabilitation therapists**						
	**Province**					
		Heilongjiang	498(88.3)	66 (11.7)	—	—
		Fujian	170 (93.9)	11(6.1)	2.363 (1.203-4.639)	.01
	**Education level**					
		High school and below	62 (76.5)	19 (23.5)	—	—
		Vocational college	271 (89.4)	32 (10.6)	2.777 (1.465-5.264)	.002
		University and above	335 (92.8)	26 (7.2)	4.415 (2.278-8.556)	<.001

^a^OR: odds ratio.

^b^Reference values.

### Benefits and Barriers Associated With Using Digital Health Services

**Figure 1:**
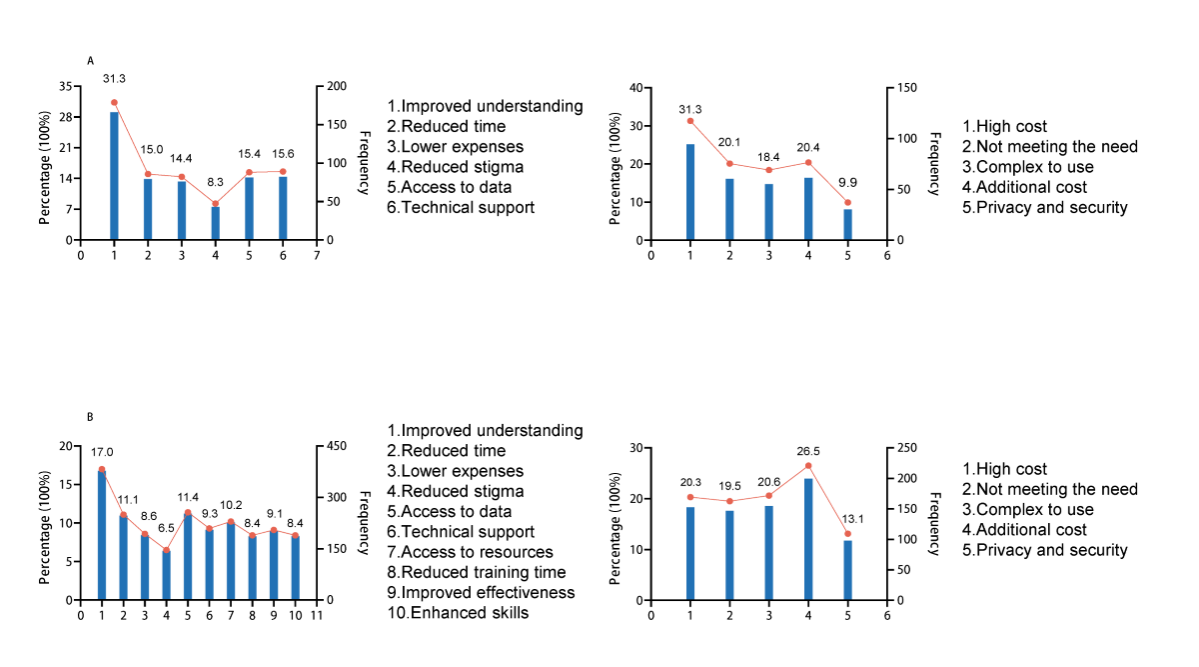
Perceived benefits and barriers associated with using digital health services among (A) parents of children with autism spectrum disorder and (B) rehabilitation therapists with autism spectrum disorder–related experiences, based on a cross-sectional survey carried out in Heilongjiang and Fujian provinces, China.

The top 3 perceived advantages of using digital health services according to parents were “Improved understanding of my child’s behavior and development,” “Technical support for rehabilitation training,” and “Easier access to data and information,” as shown in Figure 1A. In contrast, the 3 most frequently reported barriers to use of digital health services according to parents were: “High cost,” “Additional equipment is required, which increases costs,” and “Services do not fully meet the needs of families with autism,” as shown in Figure 1A.

Rehabilitation therapists reported the following top advantages of using digital health services, as shown in Figure 1B: “Improved understanding of children’s behavior and development,” “Easier access to data and information,” and “Reduced time spent on medical consultations.” Conversely, the top 3 disadvantages of using digital health services according to respiratory therapists were “Additional equipment is required, which increases costs,” “Technology is difficult or complicated to use,” and “high cost” (Figure 1B).

### Preferences for Digital Health Services

**Figure 2:**
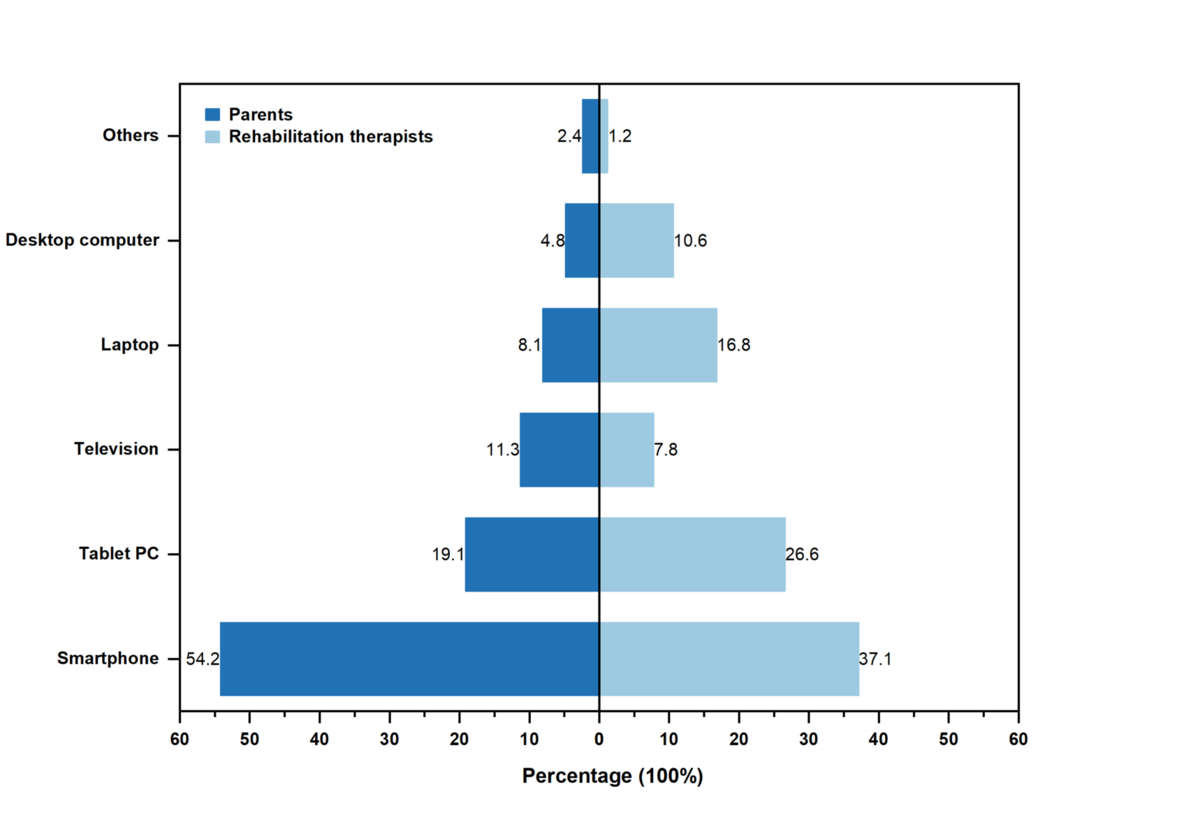
Device preferences for digital health services among parents and rehabilitation therapists caring for individuals with autism spectrum disorder based on a cross-sectional survey carried out in Heilongjiang and Fujian provinces, China.

**Figure 3:**
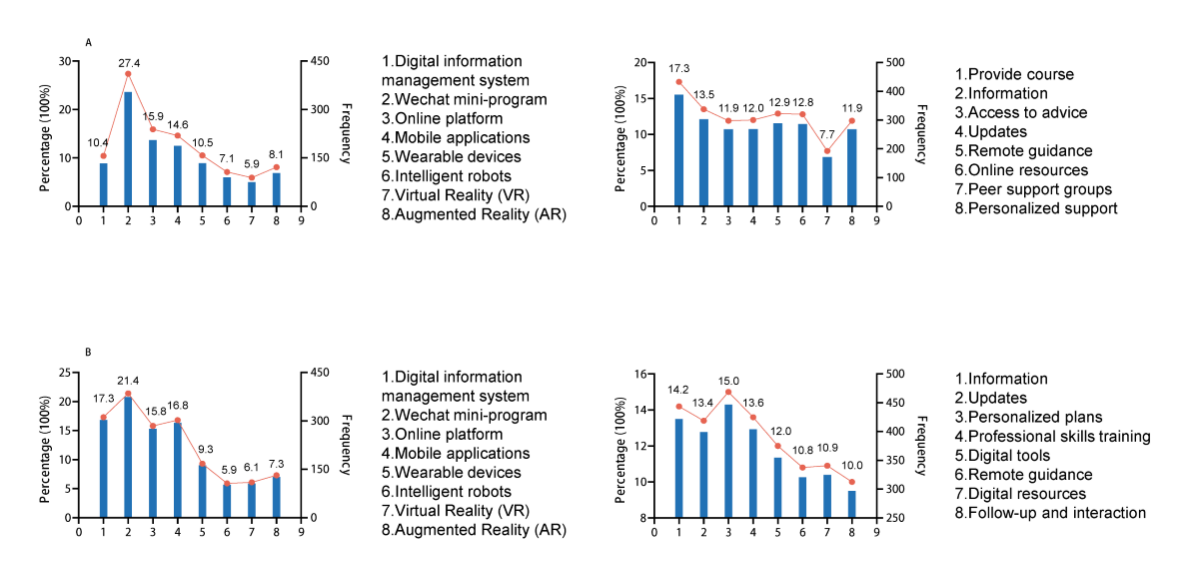
Platforms and content preferences for digital health service among (A) parents of children with autism spectrum disorder and (B) rehabilitation therapists with autism spectrum disorder–related experiences, based on a cross-sectional survey carried out in Heilongjiang and Fujian provinces, China.

Figure 2 illustrates participants’ preferences regarding devices used to access digital health services. Most study participants preferred smartphones, followed by tablet personal computers. Notably, parents had a higher preference for internet protocol television, whereas rehabilitation therapists favored laptops. Figure 3 depicts the preferred digital service platforms. Parents of children with ASD preferred using the WeChat mini-program, web-based platforms, and mobile apps. Rehabilitation therapists had higher use of the WeChat mini-program, digital information management systems, and mobile apps. Parents of children with ASD used digital health services to obtain the following: “Treatment or rehabilitation courses,” “Information about autism and related policies,” “Remote guidance for home-based interventions,” For rehabilitation therapists commonly Rehabilitation therapists used digital health services to obtain the following: “Personalized rehabilitation plans for children,” “Information about autism and related policies,” and “Professional skills training.”

## Discussion

### Principal Findings

This study provided novel insights into the current status, influencing factors, and perceived needs related to digital health service adoption among the parents of children with ASD and rehabilitation therapists in China. This cross-sectional survey revealed a low digital health service use (46/780, 5.9% among parents and 161/745, 21.6% among therapists) despite high demand (621/780, 79.6% and 671/745, 90.0% respectively). The predictors of use varied. Specifically, younger age, employment, lower household income, and delayed ASD diagnoses were associated with higher use of digital health services by parents, while male, special education background, and autism-specific training were key factors among therapists. Demand for digital health services was significantly higher in Fujian than in Heilongjiang and was positively associated with higher education levels. Both groups identified improved understanding of ASD behaviors and development, and easier access to resources as primary benefits, while high cost, additional equipment requirements, and usability issues were common barriers to digital health service use. Smartphones were the most preferred devices for digital health service use, with WeChat mini-programs and mobile apps identified as favored platforms. The preferred digital health service content for parents and therapists included rehabilitation course, remote guidance, policy information, personalized plans, and professional skills training. Despite the increasing availability of digital technologies, digital health service use remains suboptimal, with significant disparities across demographic, regional, and professional variables. These findings underscore the urgent need for more inclusive strategies to expand the reach, effectiveness, and equity of digital health in ASD care.

### Low Use Despite High Demand

Although digital health technologies have become more widely available, the adoption of digital health services among key ASD stakeholders is limited. Indeed, only 5.9% (46/780) of parents of the children with ASD and 21.6% (161/745) of rehabilitation therapists reported digital health services use, with most engaging just 1-2 times per week. These figures reflected a heavy reliance on traditional, face-to-face rehabilitation approaches, as well as limited awareness of the broader functions and benefits of digital health beyond information retrieval. Previous research has shown that parents primarily depend on direct guidance from therapists, suggesting missed opportunities for autonomous, technology-facilitated learning and support [17]. In contrast, demand for digital health services was markedly higher; 79.6% (621/780) of parents and 90.0% (671/745) of therapists expressed a need for digital health services. Our results agree with previous study findings. A study that has assessed the perspectives of patients with ASD on assistive technology in China also reported low awareness and utilization rates, despite substantial unmet demand [18]. Similarly, speech therapists regarded VR as a promising tool for children with ASD. However, even though approximately 92% of speech therapists are aware of VR, only 1.82% reported using VR in clinical practice [19]. Furthermore, a survey on smartphone apps for bipolar disorder demonstrated that even though patients expressed a strong interest in these eHealth tools, health care providers had limited adoption rates. This discrepancy might be attributable to the digital divide (eg, barriers to access) and concerns regarding lower patient engagement [20]. Taken together, these studies and our findings supported a consistent conclusion. Specifically, digital health services currently exhibit a “high demand, low adoption” phenomenon as clinical tools within the mental health domain. This gap reflects structural barriers, such as limited awareness, digital literacy, affordability, and infrastructure—rather than a lack of perceived usefulness. The COVID-19 pandemic increased general familiarity with digital tools and personal experience of using digital platforms to consult health-related information, but awareness of the specific applications in autism care is still insufficient [21]. These findings highlight the need for broader dissemination, training, and contextualized digital health promotion strategies.

### Determinants of Use and Demand

Parental engagement with digital health services was significantly associated with younger age, employment, and higher formal education, all of which are indicators aligned with prior research on digital literacy and eHealth readiness [22,23]. Interestingly, this study showed that parents with lower household incomes had higher digital health services usage, which may reflect the widespread availability and affordability of smartphones [24]. This finding is in agreement with international research findings that showed proliferation of mobile devices has resulted in greater reliance on mobile technology for accessing web-based information among socioeconomically disadvantaged groups [25]. This finding suggested that mobile technology may serve as a tool for narrowing socioeconomic gaps in access to ASD-related services. In this case, families with lower economic levels may be more eager to learn about relevant policies and obtain financial help through digital health services, or hope to reduce rehabilitation expenses and the family burden through their own learning [26]. The study also showed that parents who had been caregivers for a longer duration and whose children were diagnosed at older ages (7-12 years) had increased use of digital health services. This finding suggested that the cumulative burden of caregiving, particularly when paired with delayed diagnosis, may drive parents to seek supplemental support through digital means [27]. Late diagnosis of ASD is often correlated with prolonged uncertainty, limited early intervention, and higher stress levels, factors that increase the perceived utility of digital health services for self-education, remote consultations, and at-home interventions [28,29].

Among therapists, male, specialization in special education, and prior autism-specific training were significantly associated with higher digital health services use. Therapists with a higher level of education and those based in economically advantaged regions (Fujian province) also expressed greater demand. These findings were consistent with evidence suggesting that training and confidence in technology use influenced adoption behavior. Therapists predominantly used digital health services to improve professional skills, yet few were aware of the full potential to enhance patient outcomes. Like other research findings, rehabilitation trainers with higher levels of education and autism rehabilitation skills tended to be more inclined to understand emerging developments in the field of autism rehabilitation and improve their professional literacy [30]. Digital health platforms should go beyond information dissemination to offer interactive, personalized rehabilitation tools. Moreover, given that parents often rely on therapists for autism-related information, improving eHealth literacy among therapists may indirectly enhance parental engagement [17].

The study revealed pronounced regional disparities in the demand for digital health services, with higher demand observed in Fujian compared with Heilongjiang in both participant groups. This disparity may reflect inequities in economic development, digital infrastructure, health care access, and local policy support for technology-driven health care initiatives. Participants in Fujian generally had higher income and educational levels, factors strongly correlated with digital health engagement. These findings underscore the need for localized policy and infrastructural interventions to ensure equitable access and encourage adoption in underresourced regions [31-33].

### Perceived Benefits and Barriers

The survey results showed that parents of children with ASD and rehabilitation therapists evaluated the digital health service positively, indicating that digital health has great potential for users and room for development. Both parents and therapists recognized the potential of digital health services to improve understanding of child behavior and development, support self-monitoring, and streamline access to professional resources. However, high cost, the need for additional equipment, and usability challenges emerged as key barriers, confirming global concerns about the “digital divide”. This digital divide, rooted in socioeconomic, educational, and geographic inequalities, may offset the equity gains promised by digital health. While both high cost and additional equipment contribute to the financial burden, the basis for the impact differs. In this study, high costs stemmed from subscriptions, paid digital courses, and proprietary platform fees. These recurring expenses accumulate over time and may be especially prohibitive for families already facing significant offline care-related expenditures. In contrast, additional equipment denotes the need for specialized hardware beyond commonly owned devices, such as VR/AR systems, robotic training units, or advanced sensors. These technologies encompass substantial upfront investment and ongoing maintenance expenses, disproportionately affecting low-income families and underfunded institutions, especially in rural areas with limited internet infrastructure. Despite some promising use in geographic regions, like Shanghai, the application of advanced digital technologies, such as robotic systems and immersive environments, remains largely experimental and inaccessible in most parts of China [34]. This finding further highlights the importance of policy and institutional support in scaling such innovations. Interestingly, the observed lower income-higher use finding may seem counterintuitive but reflects the cost structure of available tools. These low-income families frequently rely on free or low-cost resources, such as open-access educational videos and policy information platforms on WeChat mini-programs, to supplement conventional rehabilitation. By reducing travel expenses, lost work hours, and direct service fees, such resources function as cost-saving measures rather than additional financial burdens. Conversely, high-cost digital services are used less often, underscoring a stratified usage pattern in which affordability influences both adoption and the type of service accessed.

### Platform and Content Preferences

Smartphones emerged as the preferred device for digital health services among both parents and therapists, which is consistent with national data and indicates that nearly all internet users in China access services via mobile devices. WeChat mini-programs and public accounts were the most used platforms due to convenience, low cost, and a wide user base. According to the China Internet Network Information Centre, the penetration rate of WeChat has exceeded 90%, with the user base surpassing 1 billion. Interestingly, both groups exhibited limited interest in advanced technologies, such as VR, AR, or AI-driven robots, which likely reflects a lack of familiarity rather than rejection. Adoption may improve with user-centered educational initiatives and exposure through pilot programs.

This study revealed a high degree of individualization regarding the specific needs of ASD stakeholders. Parents prioritized access to rehabilitation content, policy information, and remote guidance for home-based interventions, while therapists emphasized the need for individualized training programs, policy information, and professional development. Parents expected digital health services to mimic traditional service models, providing effective interventions and rehabilitation support for their children because they recognized the invaluable impact on their children’s daily lives and social interactions. In contrast, rehabilitation therapists expected that digital health services would offer customized rehabilitation programs tailored to the individual differences of children with ASD and assist in continuously improving professional competence through training, enabling therapists to better serve children with ASD and their families. These differences in user priorities highlight the necessity of tailoring digital health services content to meet the distinct needs of each stakeholder group. In addition, the perceived demand of digital health services among parents remained primarily informational and centered around obtaining policies, guidance, and self-learning materials, rather than leveraging advanced features, like VR-based therapy or teleconsultation [34]. The limited exposure to emerging digital health modalities, particularly outside large urban centers, reflects both structural limitations and insufficient promotion.

### Limitations

This study had several limitations. First, the external validity was limited. Although 2 provinces with distinct geographic and economic characteristics were selected to capture regional differences, most participants were recruited from public institutions in urban areas, with limited representation from rural or county-level settings and private rehabilitation providers. This limitation restricts the generalizability of the findings. Second, potential unmeasurable confounding factors may have affected the results. Variables, such as regional differences in digital health policy support, disparities in digital literacy between urban and rural residents, and differences between private and public institutions in pricing and technical partnerships, were not captured, yet may directly affect utilization patterns. Finally, data collection was solely quantitative, lacking the depth that qualitative methods (eg, interviews or focus groups) can provide. Future research should incorporate mixed method approaches and expand participant groups to include policy makers, developers, and health care administrators to gain a more comprehensive understanding of barriers and facilitators.

### Conclusions

Digital health services offered a promising avenue for improving access, personalization, and continuity of care in ASD care. However, our findings exposed a persistent gap between high demand and low use, driven by regional, demographic, and systemic barriers. This study is the first to systematically examine digital health service use and needs among both parents of children with ASD and rehabilitation therapists across 2 geographically and economically distinct provinces in China (Heilongjiang and Fujian). By mapping stakeholder-specific preferences, including preferred device, platform, and content, we further provide contextually tailored insights to guide localized digital health intervention design. These findings advance the field by linking user characteristics, regional disparities, and content preferences into a coherent framework for targeted policy and program development. Bridging the digital divide through inclusive policies, professional training, and public education is essential to ensure that digital innovations translate into tangible improvements in the lives of children with ASD and their families. Collaborative efforts among health care providers, educators, developers, and policy makers will be critical to the successful integration of digital health into ASD care pathways.

## Appendix

Multimedia Appendix 1 Survey on the utilization and demand for digital health services in parents of children with autism spectrum disorder.

Multimedia Appendix 2 CHERRIES checklist.

Multimedia Appendix 3 Supplementary materials.

## Abbreviations

AI: artificial intelligence
AR: augmented reality
ASD: autism spectrum disorder
CHERRIES: Checklist for Reporting Results of Internet E-Surveys
OR: odds ratio
VR: virtual reality

## Data Availability

The datasets generated or analyzed during this study are available from the corresponding author on reasonable request.
