# Supplementary material for: Regional Disparities in the Use and Demand for Digital Health Services for Autism Spectrum Disorder in China: Cross-Sectional Survey of Stakeholder Perspectives
Source: J Med Internet Res. 2025 Oct 3;27:e77157. doi: 10.2196/77157 (PMC12534761; doi:10.2196/77157)
Supplement: Multimedia Appendix 1 [file jmir_v27i1e77157_app1.docx]

**Appendix 2**

**Survey on the utilization and demand for digital health services in parents of children with autism spectrum disorder**

**Section 1: Demographic questions**

1. **What is your region of residence?**

☐Fujian

☐Heilongjiang

1. **What is your gender?**

☐Male

☐Female

1. **What is your age?**

☐20-29 years old

☐≥30 years old

1. **What is your highest level of education?**

☐University and above

☐Vocational college

☐High school and below

1. **What is your household registration type?**

☐Urban

☐Rural

1. **What is your current employment status?**

☐Employed

☐Unemployed

1. **What is your marital status?**

☐Married

☐Divorced

☐Widowed

☐Separated

1. **What is your average monthly household income?**

☐≤3000 Chinese yuan

☐>3000 Chinese yuan

1. **What is your child's average monthly rehabilitation expenses?**

☐≤3000 Chinese yuan

☐>3000 Chinese yuan

1. **What is the gender of your child with autism?**

☐Male

☐Female

1. **What is the age of your child with autism?**

☐≥13 years old

☐7-12 years old

☐4-6 years old

☐0-3 years old

1. **What is the severity of your child with autism?**

☐Mild

☐Moderate

☐Severe

☐Unclear

1. **At what age was your child diagnosed with autism?**

☐≥13 years old

☐7-12 years old

☐4-6 years old

☐0-3 years old

1. **How long has your child with autism received rehabilitation training in total?**

☐＜1 year

☐1-2 years

☐3-4 years

☐≥5 years

1. **How many years have you been caring for your child with autism?**

☐＜1 year

☐1-2 years

☐3-4 years

☐≥5 years

**Section 2: The current use and perceived needs of digital health services**

1. **Have you ever used digital health services for your child’s autism care？**

☐Yes

☐No

1. **What benefits do you associate with using digital health services? (Select all that apply)**

☐Improved understanding of my child’s behavior and development

☐Reduced time spent on medical consultations

☐Lower expenses for medical treatment and rehabilitation

☐Reduced stigma as a parent

☐Easier access to data and information

☐Technical support for rehabilitation training

1. **What barriers have you encountered when using digital health services? (Select all that apply)**

☐High cost

☐Services do not fully meet the needs of families with autism

☐Technology is difficult or complicated to use

☐Additional equipment is required, increasing costs

☐Privacy and security concerns.

1. **Which devices do you prefer for accessing digital health services? (Select all that apply)**

☐Smartphone

☐Tablet PC

☐Television

☐Laptop

☐Desktop computer

☐Others (please specify): __________

1. **What types of digital health technologies are you interested in? (Select all that apply)**

☐Digital information management system (It refers to a system that uses computers and software to effectively manage various information and data)

☐WeChat mini-program

☐Online platform (Online courses, virtual meetings, and short-video platforms such as TikTok and Kwai.)

☐Mobile applications

☐Wearable devices (An intelligent wristband capable of recognizing and recording indicators like a child's body language and emotional changes)

☐Intelligent robots (Social robots that can simulate facial expressions, produce sounds and interact with people)

☐Virtual Reality [This immersive simulation system utilizes head-mounted display technology (e.g., VR glasses/helmets) to deliver targeted skill-building interventions. Exemplified by virtual supermarket environments, it enables children to practice transactional skills and structured social interactions with simulated personnel within ecologically valid scenarios.]

☐Augmented Reality [This augmented reality (AR) system spatially overlays virtual objects or informational content onto physical environments, enabling real-time multimodal interaction via mobile devices (e.g., smartphones, tablets). Through evidence-based 3D avatar emotion recognition tasks, it enhances pediatric affective perspective-taking capacities to improve prosocial communication competency.]

1. **What content or features would you like digital health services to provide? (Select all that apply)**

☐Treatment or rehabilitation courses

☐Information about autism and related policies

☐Access to expert advice online

☐Updates on autism research and treatment guidelines

☐Remote guidance for home-based interventions

☐Online resources for rehabilitation training

☐Peer support groups for parents

☐Personalized support.

**Appendix 2**

**Survey on the utilization and demand for digital health services in autism rehabilitation therapists**

**Section 1: Demographic questions**

1. **What is your region of residence?**

☐Fujian

☐Heilongjiang

1. **What is your gender?**

☐Male

☐Female

1. **What is your age?**

☐20-29 years old

☐≥30 years old

1. **What is your highest level of education?**

☐University and above

☐Vocational college

☐High school and below

1. **What is your professional background?**

☐Special education

☐Preschool education

☐Rehabilitation medicine、Psychology and Nursing

☐Others (please specify): __________

1. **Have you received autism-specific skills training?**

☐Yes

☐No

1. **How many years of experience do you have in autism rehabilitation?**

☐＜1 year

☐1-5 years

☐6-10 years

☐≥10 years

**Section 2: The current use and perceived needs of digital health services**

1. **Have you ever used digital health services in your practice？**

☐Yes

☐No

1. **What benefits do you associate with using digital health services? (Select all that apply)**☐Improved understanding of children’s behavior and development
   ☐Reduced time spent on medical consultations
   ☐Lower expenses for children’s treatment and rehabilitation
   ☐Reduced stigma for parents
   ☐Easier access to data and information
   ☐Technical support for rehabilitation training

☐Easier and faster access to rehabilitation resources for parents
☐Reduced training time for therapists
☐Improved effectiveness and efficiency of rehabilitation
☐Enhanced professional skills for therapists

1. **What barriers have you encountered when using digital health services? (Select all that apply)**☐High cost
   ☐Services do not fully meet the needs of families with autism
   ☐Technology is difficult or complicated to use
   ☐Additional equipment is required, increasing costs
   ☐Privacy and security concerns
2. **Which devices do you prefer for accessing digital health services? (Select all that apply)**

☐Smartphone

☐Tablet PC

☐Television

☐Laptop

☐Desktop computer

☐Others (please specify): __________

1. **What types of digital health technologies are you interested in? (Select all that apply)**

☐Digital information management system (It refers to a system that uses computers and software to effectively manage various information and data)

☐WeChat mini-program

☐Online platform (Online courses, virtual meetings, and short-video platforms such as TikTok and Kwai.)

☐Mobile applications

☐Wearable devices (An intelligent wristband capable of recognizing and recording indicators like a child's body language and emotional changes)

☐Intelligent robots (Social robots that can simulate facial expressions, produce sounds and interact with people)

☐Virtual Reality [This immersive simulation system utilizes head-mounted display technology (e.g., VR glasses/helmets) to deliver targeted skill-building interventions. Exemplified by virtual supermarket environments, it enables children to practice transactional skills and structured social interactions with simulated personnel within ecologically valid scenarios.]

☐Augmented Reality [This augmented reality (AR) system spatially overlays virtual objects or informational content onto physical environments, enabling real-time multimodal interaction via mobile devices (e.g., smartphones, tablets). Through evidence-based 3D avatar emotion recognition tasks, it enhances pediatric affective perspective-taking capacities to improve prosocial communication competency.]

1. **What content or features would you like digital health services to provide? (Select all that apply)**

☐Information about autism and related policies
☐Updates on autism research and treatment guidelines
☐Personalized rehabilitation plans for children
☐Professional skills training for therapists
☐Digital tools for assessing children with autism
☐Remote guidance for families
☐Digital resources for rehabilitation training
☐Tools for follow-up and interaction with children and parents
