# Supplementary material for: Regional Disparities in the Use and Demand for Digital Health Services for Autism Spectrum Disorder in China: Cross-Sectional Survey of Stakeholder Perspectives
Source: J Med Internet Res. 2025 Oct 3;27:e77157. doi: 10.2196/77157 (PMC12534761; doi:10.2196/77157)
Supplement: Multimedia Appendix 2 [file jmir_v27i1e77157_app2.docx]

**Appendix 2:** **CHERRIES checklist**

Checklist for Reporting Results of Internet E-Surveys (CHERRIES)

| **Item category** | **Checklist item** | |
| --- | --- | --- |
| **Design** | Describe survey design | This study employed the method of purposive sampling. Participants were recruited from designated rehabilitation institutions affiliated with the Heilongjiang Disabled Persons’ Federation, the Fujian Disabled Persons’ Federation, the Rehabilitation Department of Fujian Children’s Hospital, and the Developmental Behavioral Pediatrics Department of Xiamen Maternal and Child Health Hospital—all of which specialize in pediatric developmental disorders and provide rehabilitation services for children with ASD. For parent participants, distribution occurred primarily in waiting areas of outpatient clinics or rehabilitation centers during their child’s visit; for therapists, QR codes were shared via institutional workgroups or during staff meetings. |
| **Institutional Review Board (IRB)approval and informed consent form** | IRB approval | The study protocol was approved by the Institutional Review Board of the Sixth Affiliated Hospital, Harbin Medical University, China (LC2024-066). |
|  | Informed consent | The subjects were informed of the purpose of the survey, anonymity, confidentiality, and voluntary principles before responding. |
|  | Data protection | Informed consent was obtained from participants before they answered the survey questions. All collected data were kept strictly confidential and anonymous. |
| **Developing and pretesting** | Development and testing | Data were collected via an online platform (Questionnaire Star, Changsha Ranxing Science and Technology Co., Ltd., China). Participants accessed the survey through unique QR codes embedded in electronic invitations. A structured, self-administered questionnaire was designed collaboratively by a multidisciplinary expert panel, including clinical specialists and nursing staff, to ensure relevance and comprehensive coverage of digital health domains. Content validity was verified through a preliminary pilot test. Two tailored versions of the questionnaire were developed: one for parents and one for rehabilitation professionals (see Appendix for full instrument). The questionnaire consisted of two sections |
| **Recruitment process and description of the sample having access to the questionnaire** | Open survey versus closed survey | Closed survey. Only participants who meet the following inclusion criteria can fill out this questionnaire：(1) parents/primary caregivers of children aged 0–18 years with a confirmed ASD diagnosis who could understand Chinese and complete the questionnaire independently; (2) rehabilitation therapists (including special education teachers, psychologists, and rehabilitation nurses) currently providing ASD-related intervention services, with at least 6 months of professional experience in the field. |
|  | Contact mode | The research team collaborated with each institution to identify potential participants during routine clinical visits or rehabilitation sessions. |
|  | Advertising the survey | On-site staff (including pediatricians, rehabilitation therapists, and institutional administrators) verbally informed eligible individuals about the study’s purpose, requirements, and confidentiality measures. |
| **Survey administration** | Web/E-mail | Interested participants received a unique QR code (generated using the Questionnaire Star platform) via printed flyers or direct digital sharing (e.g., through WeChat). |
|  | Context | The commercial platform specializes in publishing online questionnaires. We only used it for data collection. |
|  | Mandatory/voluntary | The survey was voluntary. |
|  | Incentives | Participation was voluntary, and no incentives were provided for survey completion. |
|  | Time/date | This study was conducted between November 2023 and February 2024 |
|  | Randomization of items or questionnaires | Question order was not randomized. |
|  | Adaptive questioning | There were adaptive questions in the questionnaire so that respondents can jump past unnecessary questions based on their answers. For example, If the subject has never used digital health services, then there is no need to answer about the form and content of the usage. |
|  | Number of Items | The most had 21 questions, and the least had 13 questions. |
|  | Number of screens (pages) | 3 screens |
|  | Completeness check | Technically, it is possible to do completeness checks before the questionnaire is submitted, and selecting a response option should be enforced. |
|  | Review step | Respondents were allowed to go 'back' in the survey. However, they were asked to confirm that the current answer was correct before submitting the questionnaire. |
| **Response rate** | Unique site visitor | N/A |
|  | View rate (Ratio of unique survey visitors/unique site visitors) | N/A |
|  | Participation rate (Ratio of unique visitors who agreed to participate/unique first survey page visitors) | N/A |
|  | Completion rate (Ratio of users who finished the survey/users who agreed to participate) | N/A |
| **Preventing multiple entries from the same individual** | Cookies used | N/A |
|  | IP check | Restrictions were placed to prevent duplicate submissions from the same IP address. |
|  | Log file analysis | N/A |
|  | Registration | Users need to log in to WeChat and fill out the questionnaire. And the same IP address can only be used to fill out the questionnaire once at a time. The survey never displayed a second time once the user had filled it in |
| **Analysis** | Handling of incomplete questionnaires | Our questionnaire requires that all the questions be filled in before submission can be made. |
|  | Questionnaires submitted with an atypical timestamp | Invalid questionnaires were excluded based on the following criteria: (a) patterned or identical responses (e.g., all “1” or sequential “1, 2, 3, 4”); (b) logical inconsistencies; or (c) completion time under 2 minutes. |
|  | Statistical correction | Avoid the deviation of results by using appropriate statistical methods. For example, descriptive statistics were presented as frequencies and percentages for categorical variables. Group differences in categorical variables were assessed using chi-square (χ²) tests. The multiple-choice question response analysis was conducted by using the multiple response analysis method. |
