# Supplementary material for: Regional Disparities in the Use and Demand for Digital Health Services for Autism Spectrum Disorder in China: Cross-Sectional Survey of Stakeholder Perspectives
Source: J Med Internet Res. 2025 Oct 3;27:e77157. doi: 10.2196/77157 (PMC12534761; doi:10.2196/77157)
Supplement: Multimedia Appendix 3 [file jmir_v27i1e77157_app3.docx]

**Appendix 3**

**Table S1** Univariate analyses for estimating determinants of digital health service utilization among parents of children with ASD, based on a cross-sectional survey conducted in Heilongjiang and Fujian provinces, China (N=780).

| **Characteristics** | **Used *N* (%)** | **Unused *N* (%)** | ***χ^2^*** | ***P* value** |
| --- | --- | --- | --- | --- |
| **Province** |  |  | 0.422 | .516 |
| Fujian | 5 (4.5) | 105 (95.5) |  |  |
| Heilongjiang | 41 (6.1) | 629 (93.9) |  |  |
| **Sex** |  |  | 0.017 | .897 |
| Male | 7 (5.6) | 117 (94.4) |  |  |
| Female | 39 (5.9) | 617 (94.1) |  |  |
| **Age** |  |  | 6.676 | .010* |
| 20-29 years old | 8 (14.8) | 46 (85.2) |  |  |
| ≥30 years old | 38 (5.2) | 688 (94.8) |  |  |
| **Education level** |  |  | 1.581 | .454 |
| University and above | 15 (7.7) | 180 (92.3) |  |  |
| Vocational college | 11 (5.7) | 183 (94.3) |  |  |
| High school and below | 20 (5.1) | 371 (94.9) |  |  |
| **Household registration** |  |  | 0.308 | .579 |
| Urban | 27 (6.3) | 400 (93.7) |  |  |
| Rural | 19 (5.4) | 334 (94.6) |  |  |
| **Employment status** |  |  | 6.855 | .009^*^ |
| Employed | 30 (8.3) | 333 (91.7) |  |  |
| Unemployed | 16 (3.8) | 401 (96.2) |  |  |
| **Marital status** |  |  | 0.412 | .901 |
| Married | 42 (6.3) | 627 (93.7) |  |  |
| Divorced | 2 (3.7) | 52 (96.3) |  |  |
| Widowed | 0 (0.0) | 8 (100.0) |  |  |
| Separated | 2 (4.1) | 47 (95.9) |  |  |
| **Monthly household income** |  |  | 5.522 | .019^*^ |
| ≤3000 Chinese yuan | 20 (9.0) | 201 (91.0) |  |  |
| >3000 Chinese yuan | 26 (4.7) | 533 (95.3) |  |  |
| **Monthly rehabilitation costs** |  |  | 2.337 | .126 |
| ≤3000 Chinese yuan | 19 (7.1) | 247 (92.9) |  |  |
| >3000 Chinese yuan | 27 (5.3) | 487 (94.7) |  |  |
| **Child’s sex** |  |  | 0.234 | .629 |
| Male | 33(5.7) | 550(94.3) |  |  |
| Female | 13(6.6)) | 184(93.4) |  |  |
| **Child’s age** |  |  | 1.103 | .776 |
| ≥13 years old | 1 (4.3) | 22 (95.7) |  |  |
| 7-12 years old | 10 (4.7) | 201 (95.3) |  |  |
| 4-6 years old | 26 (6.1) | 397 (93.9) |  |  |
| 0-3 years old | 9 (7.3) | 114 (92.7) |  |  |
| **Symptom severity** |  |  | 2.882 | .410 |
| Mild | 19 (8.1) | 217 (91.9) |  |  |
| Moderate | 15 (5.0) | 288 (95.0) |  |  |
| Severe | 6 (4.7) | 123 (95.3) |  |  |
| Unclear | 6 (5.4) | 106 (94.6) |  |  |
| **Age at diagnosis** |  |  | 4.616 | .098 |
| 7-12 years old | 1 (33.3) | 2 (66.7) |  |  |
| 4-6 years old | 14 (7.1) | 182 (92.9) |  |  |
| 0-3 years old | 31 (5.3) | 550 (94.7) |  |  |
| **Cumulative therapy duration** |  |  | 1.949 | .583 |
| ＜1 year | 7 (4.5) | 147(95.5) |  |  |
| 1-2 years | 22 (6.5) | 316(93.5) |  |  |
| 3-4 years | 9 (4.8) | 179 (95.2) |  |  |
| ≥5 years | 8 (8.0) | 92 (92.0) |  |  |
| **Years spent caregiving** |  |  | 7.084 | .069 |
| ≥5 years | 8 (4.9) | 156 (95.1) |  |  |
| 3-4 years | 19 (9.5) | 180 (90.5) |  |  |
| 1-2 year | 11 (5.5) | 188 (94.5) |  |  |
| ＜1 year | 8 (3.7) | 210 (96.3) |  |  |

*^*^P*<0.05.

**Table S2** Univariate analyses for estimating determinants of digital health service utilization among rehabilitation therapists with ASD-related experiences, based on a cross-sectional survey conducted in Heilongjiang ad Fujian provinces, China (N=745).

| **Characteristics** | **Used *N* (%)** | **Unused *N* (%)** | ***χ^2^*** | ***P* value** |
| --- | --- | --- | --- | --- |
| **Province** |  |  | 0.034 | .854 |
| Fujian | 40 (22.1) | 141 (77.9) |  |  |
| Heilongjiang | 121 (21.5) | 443 (78.5) |  |  |
| **Sex** |  |  | 7.680 | .006^*^ |
| Male | 28 (33.3) | 56 (66.7) |  |  |
| Female | 133 (20.1) | 528 (79.9) |  |  |
| **Age** |  |  | 2.063 | .151 |
| 20-29 years old | 54 (25.0) | 162 (75.0) |  |  |
| ≥30 years old | 107 (20.2) | 422 (79.8) |  |  |
| **Education level** |  |  | 4.635 | .099 |
| University and above | 83 (23.0) | 278 (77.0) |  |  |
| Vocational college | 68 (22.4) | 235 (77.6) |  |  |
| High school and below | 10 (12.3) | 71 (87.7) |  |  |
| **Specialty** |  |  | 13.235 | .004^*^ |
| Special education | 45 (32.4) | 94 (67.6) |  |  |
| Preschool education | 43 (20.2) | 170 (79.8) |  |  |
| Rehabilitation medicine、Psychology and Nursing | 49 (20.5) | 190 (79.5) |  |  |
| Others | 24 (15.6) | 130 (84.4) |  |  |
| **Autism-specific skills training** |  |  | 21.487 | ＜.001^*^ |
| Yes | 157 (24.4) | 487 (75.6) |  |  |
| No | 4 (4.0) | 97 (96.0) |  |  |
| **Years in practice** |  |  | 12.414 | .006^*^ |
| ＜1 year | 16 (11.4) | 124 (88.6) |  |  |
| 1-5 years | 90 (25.9) | 257 (74.1) |  |  |
| 6-10 years | 32 (21.3) | 118 (78.7) |  |  |
| ≥10 years | 23 (21.3) | 85 (78.7) |  |  |

*^*^P*<0.05.

**Table S3** Univariate analyses for estimating determinants of demand for digital health service among parents of children with ASD, based on a cross-sectional survey conducted in Heilongjiang ad Fujian provinces, China (N=780).

| **Characteristics** | **Demand *N* (%)** | **No demand *N* (%)** | ***χ^2^*** | ***P* value** |
| --- | --- | --- | --- | --- |
| **Province** |  |  | 10.064 | .002* |
| Fujian | 100 (90.9) | 10 (9.1) |  |  |
| Heilongjiang | 521(77.8) | 149(22.2) |  |  |
| **Sex** |  |  | 0.096 | .756 |
| Male | 100 (80.6) | 24 (19.4) |  |  |
| Female | 521 (79.4) | 135 (20.6) |  |  |
| **Age** |  |  | 0.124 | .724 |
| 20-29 years old | 44(81.5) | 10 (18.5) |  |  |
| ≥30 years old | 577 (79.5) | 149 (20.5) |  |  |
| **Education level** |  |  | 18.826 | <.001^*^ |
| University and above | 175 (89.7) | 20 (10.3) |  |  |
| Vocational college | 155 (79.9) | 39 (20.1) |  |  |
| High school and below | 291 (74.4) | 100 (25.6) |  |  |
| **Household registration** |  |  | 2.062 | .151 |
| Urban | 348 (81.5) | 79 (18.5) |  |  |
| Rural | 273 (77.3) | 80 (22.7) |  |  |
| **Employment status** |  |  | 4.569 | .033* |
| Employed | 301 (82.9) | 62 (17.1) |  |  |
| Unemployed | 320 (76.7) | 97 (23.3) |  |  |
| **Marital status** |  |  | 2.076 | .557 |
| Married | 537 (80.3) | 132 (19.7) |  |  |
| Divorced | 41 (75.9) | 13 (24.1) |  |  |
| Widowed | 7 (87.5) | 1 (12.5) |  |  |
| Separated | 36 (73.5) | 13 (26.5) |  |  |
| **Monthly household income** |  |  | 5.556 | .018* |
| ≤3000 Chinese yuan | 164 (74.2) | 57 (25.8) |  |  |
| >3000 Chinese yuan | 457 (81.8) | 102 (18.2) |  |  |
| **Monthly rehabilitation costs** |  |  | 1.173 | .279 |
| ≤3000 Chinese yuan | 206 (77.4) | 60 (22.6) |  |  |
| >3000 Chinese yuan | 415 (80.7) | 99 (19.3) |  |  |
| **Child’s sex** |  |  | 0.417 | .518 |
| Male | 461(79.1) | 122(20.9) |  |  |
| Female | 160(81.2) | 37(18.8) |  |  |
| **Child’s age** |  |  | 0.721 | .868 |
| ≥13 years old | 18 (78.3) | 5 (21.7) |  |  |
| 7-12 years old | 171 (81.0) | 40 (19.0) |  |  |
| 4-6 years old | 337 (79.7) | 86 (20.3) |  |  |
| 0-3 years old | 95 (77.2) | 28 (22.8) |  |  |
| **Symptom severity** |  |  | 6.338 | .096 |
| Mild | 184 (78.0) | 52 (22.0) |  |  |
| Moderate | 248 (81.8) | 55 (18.2) |  |  |
| Severe | 108 (83.7) | 21 (16.3) |  |  |
| Unclear | 81 (72.3) | 31 (27.7) |  |  |
| **Age at diagnosis** |  |  | 1.177 | .555 |
| 7-12 years old | 3 (100) | 0 (0.0) |  |  |
| 4-6 years old | 159 (81.1) | 37 (18.9) |  |  |
| 0-3 years old | 459 (79.0) | 122 (21.0) |  |  |
| **Cumulative therapy duration** |  |  | 2.748 | .432 |
| ＜1 year | 120 (77.9) | 34 (22.1) |  |  |
| 1-2 years | 263 (77.8) | 75 (22.2) |  |  |
| 3-4 years | 154 (81.9) | 34 (18.1) |  |  |
| ≥5 years | 84 (84.0) | 16 (16.0) |  |  |
| **Years spent caregiving** |  |  | 8.801 | .032^*^ |
| ≥5 years | 132 (80.5) | 32 (19.5) |  |  |
| 3-4 years | 164 (82.4) | 35 (17.6) |  |  |
| 1-2 year | 166 (83.4) | 33 (16.6) |  |  |
| ＜1 year | 159 (72.9) | 59(27.1) |  |  |

*^*^P*<0.05.

**Table S4** Univariate analyses for estimating determinants of demand for digital health service among rehabilitation therapists with ASD-related experiences, based on a cross-sectional survey conducted in Heilongjiang ad Fujian provinces, China (N=745).

| **Characteristics** | **Demand *N* (%)** | **No demand *N* (%)** | ***χ^2^*** | ***P* value** |
| --- | --- | --- | --- | --- |
| **Province** |  |  | 4.678 | .031* |
| Fujian | 170 (93.9) | 11(6.1) |  |  |
| Heilongjiang | 498(88.3) | 66 (11.7) |  |  |
| **Sex** |  |  | 0.252 | .616 |
| Male | 74 (88.1) | 10 (11.9) |  |  |
| Female | 594 (89.9) | 67 (10.1) |  |  |
| **Age** |  |  | 0.950 | .330 |
| 20-29 years old | 190 (88.0) | 26 (12.0) |  |  |
| ≥30 years old | 478 (90.4) | 51 (9.6) |  |  |
| **Education level** |  |  | 18.889 | ＜.001^*^ |
| University and above | 335 (92.8) | 26 (7.2) |  |  |
| Vocational college | 271 (89.4) | 32 (10.6) |  |  |
| High school and below | 62 (76.5) | 19 (23.5) |  |  |
| **Specialty** |  |  | 1.239 | .744 |
| Special education | 122 (87.8) | 17 (12.2) |  |  |
| Preschool education | 191 (89.7) | 22 (10.3) |  |  |
| Rehabilitation medicine、Psychology and Nursing | 218 (91.2) | 21 (8.8) |  |  |
| Others | 137 (89.0) | 17 (11.0) |  |  |
| **Autism-specific skills training** |  |  | 0.256 | .613 |
| Yes | 576 (89.4) | 68 (10.6) |  |  |
| No | 92 (91.1) | 9 (8.9) |  |  |
| **Years in practice** |  |  | 5.395 | .145 |
| ＜1 year | 118 (84.3) | 22 (15.7) |  |  |
| 1-5 years | 316 (91.1) | 31 (8.9) |  |  |
| 6-10 years | 135 (90.0) | 15 (10.0) |  |  |
| ≥10 years | 99 (91.7) | 9 (8.3) |  |  |

*^*^P*<0.05.
